# Microstructured Optical Fiber Sensors Embedded in a Laminate Composite for Smart Material Applications

**DOI:** 10.3390/s110302566

**Published:** 2011-02-28

**Authors:** Camille Sonnenfeld, Sanne Sulejmani, Thomas Geernaert, Sophie Eve, Nicolas Lammens, Geert Luyckx, Eli Voet, Joris Degrieck, Waclaw Urbanczyk, Pawel Mergo, Martin Becker, Hartmut Bartelt, Francis Berghmans, Hugo Thienpont

**Affiliations:** 1 Vrije Universiteit Brussel, Pleinlaan 2, B-1050 Brussel, Belgium; E-Mails: ssulejma@vub.ac.be (S.S.); tgeernae@vub.ac.be (T.G.); fberghma@vub.ac.be (F.B.); hthienpo@vub.ac.be (H.T.); 2 CRISMAT CNRT-Matériaux/ENSICAEN, UMR 6508, UMS 3318, 6, Boulevard du Maréchal Juin, 14050 Caen Cedex 04, France; E-Mail: sophie.eve@ensicaen.fr; 3 Universiteit Gent, Sint-Pietersnieuwstraat 41, B-9000 Gent, Belgium; E-Mails: Nicolas.Lammens@ugent.be (N.L); Geert.luyckx@ugent.be (G.L); evoet@fos-s.com (E.V.); Joris.Degrieck@UGent.be (J.D.);; 4 Wroclaw University of Technology, Wybrzeze Wyspianskiego 27, 50-370 Wroclaw, Poland; E-Mail: Waclaw.Urbanczyk@pwr.wroc.pl; 5 Marie Curie-Sklodowska University, Pl. M. Curie-Sklodowskiej 3, 20-031 Lublin, Poland; E-Mail: pawel.mergo@poczta.umcs.lublin.pl; 6 Institute of Photonic Technology, Albert-Einstein-Straße 9, D-07745 Jena, Germany; E-Mails: martin.becker@ipht-jena.de (M.B.); hartmut.bartelt@ipht-jena.de (H.B.)

**Keywords:** Fiber Bragg grating, carbon fiber reinforced polymer, strain field monitoring

## Abstract

Fiber Bragg gratings written in highly birefringent microstructured optical fiber with a dedicated design are embedded in a composite fiber-reinforced polymer. The Bragg peak wavelength shifts are measured under controlled axial and transversal strain and during thermal cycling of the composite sample. We obtain a sensitivity to transversal strain that exceeds values reported earlier in literature by one order of magnitude. Our results evidence the relevance of using microstructured optical fibers for structural integrity monitoring of composite material structures.

## 1. Introduction

Monitoring the internal strain state of fiber-reinforced polymer materials has become an important issue since in-service strain monitoring of civil engineering and aeronautic structures can lead to improved safety and better control over costs [1]. Fiber Bragg grating (FBG) based sensors are excellent candidates for that purpose as they can be embedded in different materials for smart structure applications. These sensors combine many advantages over conventional electrical sensor configurations, such as for example their small size, their immunity to electromagnetic interference, their multiplexing capabilities and self-referencing ability together with an often linear response that is encoded in the change of their reflected resonance wavelength.

To monitor the structural health of composite fiber-reinforced polymer (CFRP), one ideally needs a complete mapping of the internal strain field of the material using multi-axial strain sensors. One also needs to distinguish between the strain occurring in the axial and in the transverse directions. Monitoring the strain in the transverse direction of a CFRP in a laminated configuration is indeed an essential issue. In such structures reinforcement fibers enhance the mechanical strength of the composite in the plane of the structure, but they suffer from fragility in the transverse direction. One therefore needs to develop a system that is able to sense the out-of-the plane strains in composite laminates.

The possibility of embedding optical fibers in anisotropic materials has already been demonstrated [2]. Nevertheless most of the work published so far has focused on axial load and temperature variations, but did not investigate in detail the out-of-plane strain which is a key parameter in the structural integrity of anisotropic materials. The sensitivity to transversal strain also remained considerably lower than the sensitivity to axial strain [3,4]. In recent years substantial research efforts also attempted at developing multi-parameter FBG sensors. Several reports described the characterization of gratings subjected to transversal strains [5,6]. The Bragg gratings were written either in polarization maintaining-fibers [4,7,8] or in microstructured optical fiber [9]. However distinguishing between transversal and axial strain and correcting for temperature cross-sensitivity still required to use a second grating [8].

Several studies indeed relied on two FBGs written in the same fiber: the first grating is sensitive to all external perturbations (strain and temperature) while the second grating remains isolated from (part of) the external mechanical strain. This approach allows precisely defining the influence of each perturbation [10,11] but the presence of the capillary used to isolate the second grating from mechanical load disturbs the structure under test.

In our work we demonstrate a new optical fiber sensor specifically designed to identify transverse strain components in a CFRP with a much higher sensitivity than ever achieved before, and with a response that is independent of temperature variations. To do so we use FBGs inscribed in a highly birefringent microstructured optical fiber (MOF). Our paper is structured as follows. In the next section we summarize the theory of FBGs and we characterize the Bragg grating inscribed in the MOF. In Section 3 we theoretically and mechanically characterize the response of the bare free standing optical fiber to a transverse line load. In the fourth section we describe the embedding procedure and we assess the behavior of the embedded fiber under several loading conditions (temperature, axial and transversal tests). We eventually compare our results with those already reported in literature. Finally we conclude on the possible use of integrated MOFs for precisely monitoring transverse strain fields in laminated composite structures.

## 2. Description of the Microstructured Optical Fiber and Characteristics of the Bragg Grating

### 2.1. Fiber Bragg Grating Sensor Theory

A fiber Bragg grating is a periodic modulation of the refractive index of the fiber core, extended over a limited length of the fiber (typically a few mm). A FBG acts as a wavelength selective filter for light traveling along the fiber: it reflects light with a spectrum centered on the Bragg wavelength λ_B_, which is given by the following equation:

(1) $$\lambda_{B}=2.\;n_{\mathrm{eff}}.\Lambda$$

where Λ is the grating period and *n_eff_* is the mean effective refractive index in the core region [12]. In a birefringent fiber two orthogonally polarized modes can propagate along the fiber with different phase velocities. A Bragg grating in such a fiber will return two reflection peaks (at wavelengths λ_B1_ and λ_B2_) corresponding to the two orthogonally polarized modes travelling along respectively the slow and fast axis of the optical fiber. The spectral distance Δλ_B_ between these two peaks, to which we will refer as the wavelength separation, is proportional to the phase modal birefringence B:

(2) $${\Delta \lambda }_{B}=2.\Lambda .B$$

(3) $$B=n_{1}=n_{2}$$

where n_1_ and n_2_ are the effective indices of the two orthogonally polarized modes. Both the effective indices and the grating period will be affected by temperature and by applied mechanical strain. To determine the resulting Bragg wavelength shift of FBGs written in mechanically anisotropic optical fibers, Kim *et al.* [13] made the assumption that most of the energy of the fundamental mode propagating in the fiber is contained within the core and hence the principal strains at the centre of the fiber are sufficient to determine this wavelength shift. Under this approximation and when assuming constant temperature, the wavelength shifts can be derived from the total strain field present in the center of the fiber core as:

(4) $$\frac{{\Delta \lambda }_{B,1}}{\lambda_{B,1}}={ɛ}_{3}-\frac{1}{2}n_{1}^{2}\left[p_{11}{ɛ}_{1}+p_{12}\left({ɛ}_{2}+{ɛ}_{3}\right)\right]$$

(5) $$\frac{{\Delta \lambda }_{B,2}}{\lambda_{B,2}}={ɛ}_{3}-\frac{1}{2}n_{2}^{2}\left[p_{11}{ɛ}_{2}+p_{12}\left({ɛ}_{1}+{ɛ}_{3}\right)\right]$$

where ɛ_1_, ɛ_2_ and ɛ_3_ are the principal strain components along the axes of the fiber (ɛ_3_ refers to the axial direction), p_11_ and p_12_ stand for the strain-optic coefficients [14], λ_B1,2_ denote the initial unstrained wavelengths of the Bragg peaks for each polarization mode and Δλ_B,1,2_ are the Bragg wavelength peak shifts. By applying mechanical strain to the grating, the phase modal birefringence is modified, which in turn induces a measurable variation of the wavelength separation.

### 2.2. Fiber Microstructure Topology and Description of the Bragg Grating

**Figure 1.**
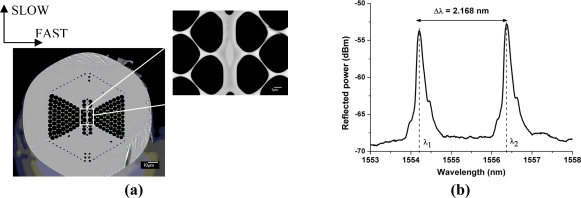
**(a)** Scanning electron microscope micrograph of a cross-section of the studied MOF and close up of the core region. **(b)** Bragg grating spectrum inscribed in the fiber core.

In this report we rely on a microstructured optical fiber. The cross-section of the silica fiber contains a doped core with a GeO_2_ concentration of 7.4 mol% and a distribution of micro air-holes running along the fiber length [Figure 1(a)]. It is already well known that by modifying the air-hole microstructure, i.e. by changing the diameter, or the distance between the air-holes and their location in the fiber cross-section, one can tailor the guiding properties of the fiber for specific applications and enhance its sensitivity to particular physical quantities [15]. The air-hole topology of the MOF under consideration has already demonstrated a high sensitivity to hydrostatic pressure [16]. Here we focus on its high sensitivity to transversal loading. Moreover, the phase modal birefringence in this MOF is inherently insensitive to temperature as reported in [16] which helps avoiding complex temperature compensation systems.

The function of the germanium doped fiber core is to allow using conventional ultraviolet fiber Bragg grating inscription methods. The FBGs were written with pulses of UV radiation originating from a Kr:F laser operating at 248 nm and with an interferometric Talbot system [17]. The writing of Bragg gratings in the germanium doped core of MOFs using this inscription technique has already been reported in [18]. The gratings are 5 mm in length with an averaged linewidth of 201 pm. The resulting Bragg spectrum shown in Figure 1(b) exhibits two clearly identified Bragg peaks. The peak separation is about 2 nm at λ = 1,550 nm which corresponds to a modal birefringence of about 2 × 10^−3^. This value is almost one order of magnitude larger than the values for conventional birefringent optical fibers, e.g., as reported in [19].

## 3. Simulations and Experiments for the Bare Microstructured Optical Fiber

The sensing potential of a FBG in the proposed MOF can already be demonstrated by characterizing its response to a transversal line load. The experiments were made by compressing the grating between a metal and a glass plate in a dedicated test bench for the calibration of FBG-based sensors [20]. We applied a transversal line load of 0.2 N/mm to the fiber and monitored the wavelength peak variation using an amplified spontaneous emission source, a circulator and a commercially available FBG interrogator with a peak wavelength detection resolution of 1 pm.

**Figure 2.**
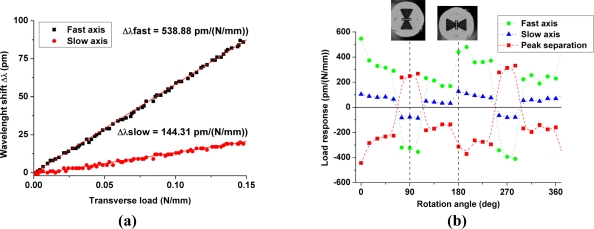
**(a)** Wavelength shift of the Bragg peaks *versus* transversal line load for one particular fiber orientation. The red solid lines represent the linear fit. **(b)** Transverse line load sensitivity plotted against angle of rotation for FBG in the fiber. The dashed vertical lines indicate the maximum values.

Since the air-hole topology is highly asymmetric, the transversal line load sensitivity changes with the angular orientation of the applied force with respect to the fiber cross-section. The grating sensitivity is determined as the slope of a linear fit of the Bragg peak wavelength shift *versus* the applied transversal line load for various angular orientations in steps of 15° [Figure 2(a)]. The typical values of the R^2^ coefficients of the linear fits were above 0.995. The fiber response according to the force orientation exhibits, as expected, a periodic behavior [Figure 2(b)]. We can point out the differences in the fiber response at 0° and at 360° while the fiber orientations are theoretically identical. These different values are most likely due to the difficult control of the fiber orientation and hence to its uncertainty during the experiments. The highest sensitivity of about −370 pm/(N/mm) is obtained when the load is applied along the slow axis of the fiber. The large side microstructure is designed to guide the applied load along this eigenaxis to confine the stress into the core, which increases the material birefringence and hence the sensitivity. We can compare our sensitivity to results published in literature. It is twice as large as that obtained using conventional highly birefringent (HB) fibers such as bow-tie fiber [160 pm/(N/mm) [19]]. In 2007 the sensing characteristics of a 220 μm diameter side-hole fiber were investigated [7]. The Bragg peak separation sensitivity reached 176 pm/(N/mm) which is half the value found in our experiments. In 2009 the Bragg peak separation sensitivity of a simple microstructured fiber tested in diametrical compression was reported to be about 100 pm/(N/mm) [20].

A numerical simulation of the FBG sensitivity to transversal line load was performed with the commercially available COMSOL Multiphysics software in a two-dimensional finite element model [16,20,21]. The exact dimensions of the simulated fiber have been extracted from a scanning electron microscope image of the fabricated fiber. The simulation combines mechanical calculations using the plane strain approximation and optical calculations where a full-vector perpendicular wave representation is used to solve for the optical modes. The mechanical calculations attempt at determining the stress distribution in the fiber which is subsequently used in the optical calculations to find the effective refractive indices of the fundamental optical modes of the fiber.

**Figure 3.**
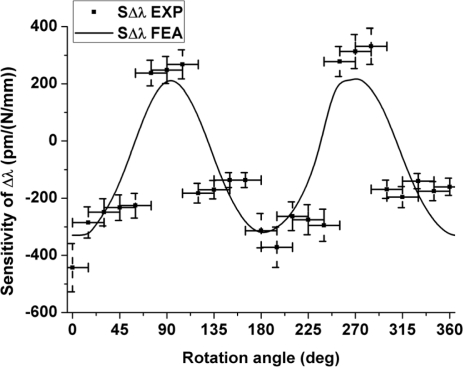
Comparison between experimental (EXP) and simulated values (FEA): sensitivity of the wavelength separation *versus* fiber orientation.

The results for the maximum and minimum sensitivity obtained from the finite element model show a reasonable agreement with the experimental results (Figure 3). The deviations are most likely due to the uncertainty on the angular orientation of the optical fiber. As can be seen in Figure 1(a) the outer shape of the cladding is not perfectly circular, which induces deviations in orientation between the portion of the fiber held in the rotation stages and the portion clamped between the load applying plates. The flattened shape of the outer cladding is therefore responsible for the flattened portions in the angular sensitivity of the fiber observed in Figure 3. The simulation nevertheless confirms that the sensitivity is maximal when the load is applied along the slow axis of the fiber. We therefore chose this orientation to embed the fibers into the composite material samples to reach the highest sensitivity in the transversal direction of the samples.

## 4. Response of the Embedded FBGs for Various Loading Conditions

The MOFs were embedded in composite materials in the previously defined orientation. Three different types of loads were applied to the test specimens to compare the sensor responses: axial strain, transversal strain and temperature tests were carried out.

### 4.1. Embedding Process and Verifications

The FBGs have been embedded at mid-thickness of a lay-up of 16 carbon fiber epoxy unidirectional prepreg layers with a total thickness of 1.54 mm (M18/M55J material). The fiber coatings have been removed using an optical fiber mechanical stripper after a chemical treatment over a few centimeters at the location of the Bragg grating. The composite samples are processed in an autoclave using the vacuum bagging technique, in which a temperature and pressure cycle is applied to the composite. The test specimens have a symmetric lay-up ([90_2_, 0_2_]_2S_) and the optical fibers are oriented with their fast axes parallel to the surface of the composite coupon. Visual inspection of the fiber orientation (after the loading experiments) revealed a very low deviation from the desired orientation of about ±3°.

**Figure 4.**
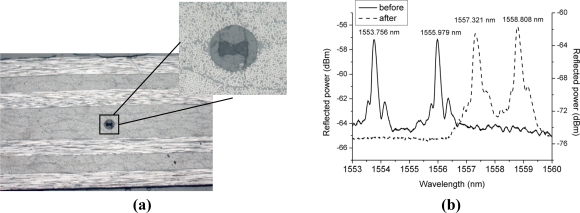
**(a)** Transversal cross-section of the composite samples at the grating location. **(b)** Spectrum of the Bragg grating sensor before and after embedding in composite materials.

The optical fibers have much greater dimensions than the carbon reinforcement fibers of the plies. Embedding the fibers could therefore result in a geometric distortion of the surrounding fibers by creating resin-rich pockets [22]. To avoid that problem the optical fibers are integrated in the same direction as the reinforcement fibers. As seen in Figure 4(a), the optical fiber is homogeneously surrounded by resin and reinforcement fibers which should have the least detrimental consequences on the structural integrity of the host material. After embedding, the optical spectrum is still visible and both peaks are clearly distinguishable, but we notice a shift of both peaks to higher wavelengths [Figure 4(b)]. This peak shift is the result of the residual strains occurring during the production process of the laminate and arising from the mismatch in material properties between the reinforcement fibers and the resin. This was also shown in [3], where the curing process of a composite laminate was monitored by a FBG inscribed in a conventional HB optical fiber to investigate the possibility to monitor the different effects of axial and transversal stress and to determine the residual stress.

### 4.2. Temperature Tests

**Figure 5.**
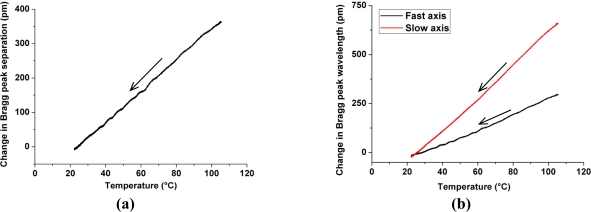
Responses of the embedded FBGs during the cooling phase; the temperature variation is indicated with arrows. **(a)** Change in Bragg peak separation *versus* temperature. **(b)** Change in Bragg peak wavelength *versus* temperature.

The samples were submitted to a thermal cycle in a dedicated climate chamber; the temperature was increased from ambient to 120 °C and then the samples were allowed to cool down slowly over a period of 10 hours. The embedded FBGs responses were monitored during the cooling phase which is considered as a quasi-static temperature variation. The temperature sensitivities of both peaks were determined by linear fits of the curves [Figure 5(b)] with R^2^ coefficients above 0.994. The temperature responses of the slow and fast axes are respectively 8.02 pm/°C and 3.61 pm/°C. The different sensitivity of both modes is due to the release of the asymmetric strains occurring in the composite sample during its cooling to ambient temperature. The temperature sensitivity of the Bragg peak separation is about of 4.41 pm/°C [Figure 5(a)].

The temperature sensitivity is not inherent to the fiber, but should be understood as the result of the modified stress/strain state induced by a temperature change inside the composite sample. We have indeed already demonstrated that this microstructure is intrinsically insensitive to temperature [16] and hence the wavelength changes occurring during the thermal cycles do come from strain of the surrounding material. Moreover for the bare optical fiber the Bragg peak separation sensitivity is about −0.07 pm/°C which is more than 60 times lower than the Bragg peak separation sensitivity for the FBG embedded in composite laminate. The temperature sensitivity is induced by the outer strain and therefore the proposed sensor holds potential to evaluate process-induced residual strain in composite samples and therefore to identify the initial internal state of a composite structure.

### 4.3. Axial Strain Test

**Figure 6.**
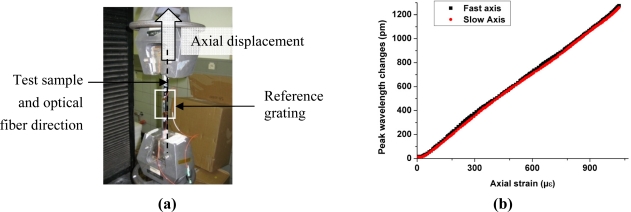
**(a)** Axial load set-up. **(b)** Peak wavelength changes *versus* axial strain.

The samples were loaded in the axial direction corresponding to the integrated optical fiber direction and the wavelength changes were monitored at the same time. The axial strain applied to the samples is measured by a reference grating glued on top of the sample at the exact location of the Bragg grating of interest [Figure 6(a)]. A maximum strain of 1,000 μɛ is applied to the laminate at a rate of 200 μɛ/min.

Figure 6(b) shows that both peaks react in the same way and in a linear manner. Consequently the peak separation of the sensor is almost insensitive to axial strain for both samples (Table 1) and the fast and slow axes sensitivities are very close to those of the bare fiber (about 1.18 pm/μɛ).

### 4.4. Transverse Strain Test

**Figure 7.**
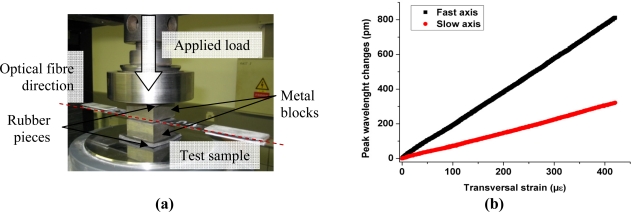
**(a)** Transverse load set-up. **(b)** Peak wavelength changes *versus* transversal strain.

Transverse strain tests were performed on the sensors to characterize the out-of-plane laminate response. The specimens are compressed between two metal blocks with an increasing load of maximum 20 kN which corresponds to about 25 MPa (8 cm^2^ contact surface). To ensure a homogenous pressure on the composite, two rubber slices are placed between the metal blocks and the load applying surfaces [Figure 7(a)]. The wavelength of the peaks and the load are recorded continuously (read-out frequency 1 Hz) during the test. By approximating the Young modulus of the laminate in the load direction by 6 GPa, we can estimate the transversal strain applied to the samples.

Figure 7(b) shows the linear behavior of the peak wavelength *versus* the transversal strain applied, from which we determine the response of the sensors (Table 2). As for the bare fiber calibration, this experiment shows that when the optical fiber is loaded along its slow axis, its response is much more important for the fast axis than for the slow axis; in this case the difference in sensitivity is more than 50%. As shown in Figure 1(a) the fiber microstructure is highly asymmetric and presents much more side-holes along the fast axis. Therefore, the stresses along the major diameter of the cladding are larger than those created along the minor diameter direction, and hence the transverse sensitivity along the fast axis of the fiber is higher compared to the slow axis.

The specific behavior of the sensors can hence be understood as follows. When the fiber is non-uniformly loaded at constant temperature, one can de-multiplex the loads acting on the optical fiber:

- a Bragg peak separation change is induced by transversal strains, because the axial strain sensitivity of the peak separation has been shown to be zero;
- identical changes in the peak wavelengths will refer primarily to axial strains. The sensitivities of the slow and fast axis for axial strain are 5 to 10 times larger than for transversal strain.

We can now assess in how much more sensitive our sensor is compared to FBGs in conventional HB optical fibers and in a MOF with another type of microstructure.

### 4.5. Comparison of the Sensor Response with Previous Studies

**Figure 8.**
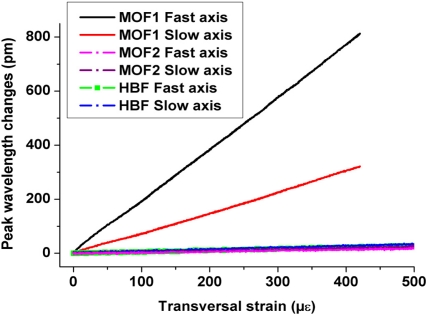
Transversal strain response of a conventional HB fiber of bow-tie type (HBF) and of the MOF2 embedded in unidirectional laminate compared with the response of MOF1 integrated in a cross-ply configuration (the sensitivity plots of MOF2 and HBF are overlaying each other).

In [23] the transversal strain response of sensors was determined for conventional bow-tie type HB optical fiber and for another MOF structure (referred to here as MOF2) both embedded in a unidirectional laminate. Figure 8 shows that the proposed MOF (referred to as MOF1) demonstrates a much higher response to transversal strain than the two other FBGs. The transversal Bragg separation sensitivity reaches about −0.16 pm/μɛ in the case of the MOF studied here; which is one order of magnitude higher than that of the conventional HB fiber and of MOF2. However the laminate composite in [23] refers to a unidirectional configuration and one therefore needs to verify how large the composite configuration influences the sensor response.

To determine how important the laminate configuration is we perform numerical simulations of the transversal strain tests. We first validate the simulations by comparing the numerical and experimental response of MOF1 and then we model the behavior of MOF2 embedded in the cross-ply configuration.

**Figure 9.**
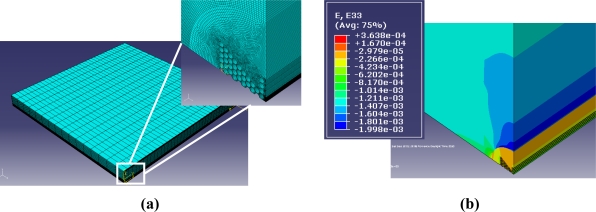
**(a)** 1/8 of the laminate structure meshed and close-up of MOF1. **(b)** Strain in the out-of-plane direction for MOF1.

The modeling of the laminate behavior submitted to transversal pressure is carried out with the commercially available finite element software ABAQUS. The goal is to link the strain in the fiber core to the strain in the laminate composite using Equations (4) and (5). The strain values used in these equations are an average of the principal strains in the entire core region all along the grating length; averaging the strain allows avoiding stress non-uniformity coming from the air-hole microstructure. We assume in the analysis that the principal strain axes and the optical axes of the anisotropic fiber overlap. We assume that the bonding between the optical fiber and the host material is perfect, the deformations are considered small and both materials behave elastically. The composite laminate model, illustrated in Figure 9(a), consists of 100,500 linear hexahedral elements of type C3D8R. The mesh is refined in the vicinity of the optical fiber. For symmetry reasons, only one eighth of the specimen is represented and specific boundary conditions are applied. The top surface is submitted to a linearly increased load of maximum 25 MPa. The simulated length is 20 mm which ensures us to be far away from the end facets and hence we avoid boundary effects.

**Figure 10.**
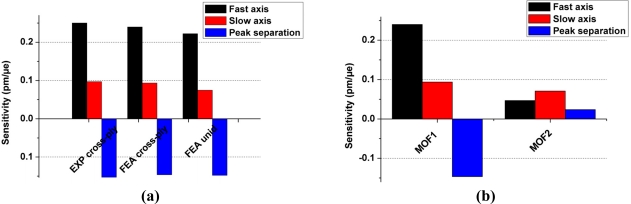
Comparison between the composite configurations. **(a)** MOF1 embedded in cross-ply laminate (experimental (EXP) and modeling (FEA)) and unidirectional laminate (modeling). **(b)** MOF1 and MOF2 in cross-ply laminate (modeling results).

Figure 10 summarizes the results of the finite element analysis (FEA). Figure 10(a) first evidences the excellent agreement between simulations and experiments for MOF1 embedded in a cross-ply laminated composite, with a relative error of only 4.5 %. This validates our model and FEA approach. Second, the model suggests that when the same MOF is embedded within a unidirectional laminated composite the sensitivity to applied load is slightly reduced by approximately 3.4% [Figure 10(a)]. This result indicates that the composite lay-up does not play a major role in the transversal sensor response. Finally, the simulations again reveal the substantially improved sensitivity of MOF1 with a factor of 6 compared to MOF2 when both fiber types are embedded within a composite with similar lay-up [Figure 10(b)].

## 5. Conclusions

We have embedded fiber Bragg gratings written in specially designed highly birefringent microstructured optical fiber in laminate fiber reinforced composite material samples, with the intention to accurately monitor the transverse strain components inside composite materials. The sensor response is encoded in the spectral distance between the two reflection peaks of the fiber Bragg grating. The gratings were successfully integrated within the composite panels without compromising the mechanical integrity of the material and the optical properties of the grating. The embedded Bragg gratings exhibit a substantial improvement in sensitivity to transversal strain and a low sensitivity to temperature changes compared to values reported earlier in literature. The embedded sensor sensitivity to temperature was 4.41 pm/°C, the sensitivity of the peak separation to transversal strain was 0.16 pm/μɛ, while the sensitivity of the peak separation to axial strain remained negligible. Transversal strains can therefore be identified with resolutions down to about 10 μɛ using commercial fiber Bragg grating interrogation equipment. Furthermore and owing to the selective response of our sensor and to the bare sensor temperature sensitivity of 0.07 pm/°C, composite curing and residual strains can be monitored as well. This evidences the potential of using dedicated microstructured optical fibers in the field of structural health monitoring.

## Figures and Tables

**Table 1.** Axial strain sensitivity of FBGs sensors embedded in [0_2_, 90_2_]_2S_ lay-up composite.

**pm/μɛ**	**Average on sample tested**

Fast axis	1.22 ± 0.03
Slow axis	1.21 ± 0.02
Peak separation	−0.01 ± 0.01

**Table 2.** Transversal strain sensitivity of the MOF.

**pm/μɛ**	**Average on the tested samples**

Fast axis	0.26 ± 0.05
Slow axis	0.10 ± 0.02
Peak separation	−0.16 ± 0.03
